# Injury in Starting and Replacement Players from Five Professional Men’s Rugby Unions

**DOI:** 10.1007/s40279-024-02014-3

**Published:** 2024-04-12

**Authors:** Simon P. Roberts, Keith A. Stokes, Sean Williams, Stephen W. West, Simon Kemp, Matt Cross, Isabel S. Moore, Charlotte Leah Bitchell, Prabhat Mathema, Ken Quarrie, Warren McDonald, Lauren Fortington, Eduardo Rubio del Castillo, Clint Readhead, Nicola Sewry, Éanna Falvey, Ross Tucker

**Affiliations:** 1 Centre for Health, and Injury and Illness Prevention in Sport, Department for Health, University of Bath, Bath, UK; 2 UK Collaborating Centre on Injury and Illness Prevention in Sport (UKCCIIS), University of Bath, Bath, UK; 3 Rugby Football Union, Twickenham, UK; 4 Sport Injury Prevention Research Centre, Faculty of Kinesiology, University of Calgary, Calgary, Alberta Canada; 5 London School of Hygiene and Tropical Medicine, London, UK; 6 Premiership Rugby, Twickenham, UK; 7 Cardiff School of Sport and Health Sciences, Cardiff Metropolitan University-Cyncoed Campus, Cardiff, UK; 8 Medical Department, Welsh Rugby Union, Cardiff, UK; 9 New Zealand Rugby, Wellington, New Zealand; 10 Sports Performance Research Institute, Faculty of Health and Environmental Sciences, Auckland University of Technology, Auckland, New Zealand; 11 Rugby Australia, Moore Park, NSW Australia; 12 University of Canberra, Bruce, ACT Australia; 13 School of Medical and Health Sciences, Edith Cowan University, Joondalup, WA Australia; 14 South African Rugby Union, Cape Town, South Africa; 15 Sport Exercise Medicine and Lifestyle Institute (SEMLI), Faculty of Health Sciences, University of Pretoria, Pretoria, South Africa; 16 IOC Research Centre South Africa, Cape Town, South Africa; 17 World Rugby Limited, Dublin, Ireland; 18 Carnegie Applied Rugby Research (CARR) Centre, Carnegie School of Sport, Leeds Beckett University, Leeds, UK

## Abstract

**Objectives:**

The aim of this study was to compare the incidence, severity, and burden of injury in starting and replacement players from professional men’s teams of five rugby unions.

**Methods:**

Match injuries of greater than 24 h time-loss (including data on the severity, match quarter, event, body region) and player minutes of match exposure data were collated for all starting and replacement players in the men’s English Premiership, Welsh Pro14 (both 2016/17–2018/19 seasons), and Australian, New Zealand, and South African Super Rugby (all 2016–2018 seasons) teams. Injury incidences and mean injury burden (incidence × days missed) were calculated, and rate ratios (RRs) (95% confidence intervals [CIs]) were used to compare injury incidence and burden between starting (reference group) and replacement players.

**Results:**

Overall injury incidence was not different between starters and replacements for all injuries (RR = 0.98, 95% CI 0.88–1.10), nor for concussions (RR = 0.85; 95% CI 0.66–1.11). Mean injury burden was higher for replacement players (RR = 1.31, 95% CI 1.17–1.46). Replacement injury incidence was lower than the starters in the third (RR = 0.68, 95% CI 0.51–0.92) and fourth (RR = 0.78, 95% CI 0.67–0.92) match quarters. Injury incidence was not different between starters and replacements for any match event or body region, but compared with starters, replacements’ injury burden was higher in lower limbs (RR = 1.24, 95% CI 1.05–1.46) and in the tackled player (RR = 1.30, 95% CI 1.01–1.66).

**Conclusion:**

This study demonstrated a lower injury incidence in replacement players compared with starters in the second half of matches, with a higher injury burden for replacement players due to higher mean injury severity.

**Supplementary Information:**

The online version contains supplementary material available at 10.1007/s40279-024-02014-3.

## Key Points

**Table:** 

The number of match minutes or total number of replacements used by one team did not impact on the number or burden of injuries sustained by the opposing team.
Injury incidence rates over the course of an entire match were not different between replacement and starting players, but incidence rates were lower for replacements compared with starters in the second half of matches.
This study provides objective data on which the governing body may make informed decisions with respect to starting players versus replacement players, but the current results do not support changes to the current laws on the use of replacements in the game.

## Introduction

Professional rugby union (hereafter ‘rugby’) is characterised by bouts of high-intensity running and physical contact between players [1, 2], resulting in a high injury incidence rate [3] and concussion incidence rate [4, 5]. In the professional era, the permitted use of replacements in professional matches has evolved. In 1996, six replacements were permitted, increasing to seven in 1997 and then to eight since 2014. Each team now comprises a squad of 23 players: 15 starters and eight replacements. Replacements may join the match permanently to replace an injured player or as a tactical replacement, or temporarily in the case of a blood injury or a Head Injury Assessment (HIA) (when the player being treated or assessed subsequently rejoins the match) [6].

Some former players have suggested in an open letter to World Rugby that in professional rugby, the introduction of replacements may increase the number of physical interactions between unfatigued replacements and fatigued starting players, thus increasing the injury risk to starting players and contributing towards overall injury incidence [7, 8]. While there is evidence that injury incidence rates in professional rugby match play are higher in the second half of matches compared with the first half [9], and notably in the third quarter [10], it may be only inferred that this is a result of player fatigue and does not account for starters and replacements. Indeed, few studies have investigated injuries in starting and replacement players in team sports. One study showed that Australian football players who were permitted to have a high number of rest periods, due to regular match interchanges, sustained lower rates of hamstring injuries [11], while in amateur rugby league, the introduction of fewer game player interchanges resulted in a lower injury risk [12]. The only available data relating to injury incidence in rugby starters and replacements was from 2002 to 2004, when a maximum of seven replacements were permitted, showing a higher injury incidence for starting players (114 [101–126] per 1000 h) compared with replacements (87 [66–108] per 1000 h) in the final match quarter [13].

Governing bodies have been urged to review the permitted number of replacements [7], but there is no recent empirical evidence pertaining to the injury risk of starting and replacement players to inform any potential changes in practice. Given the notion that injury risk for a team may be in part related to the number of interactions with replacements in the opposing team, the primary aim was to determine whether there was an association between the number of replacements and replacement minutes used by one team in a match and the number of injuries to the opposing team. The secondary aim of this study was to determine whether the incidence, severity, and mean burden (the product of incidence and severity) of injuries differed between starting and replacement players in professional men’s rugby.

## Methods

### Setting

This was a cross-sectional study, incorporating a combined analysis of three seasons of data from men’s professional rugby teams in England (Premiership Rugby teams for seasons 2016–2017 to 2018–2019), Wales (Pro14 Regional teams for seasons 2016–2017 to 2018–2019), and New Zealand, Australia, and South Africa (Super Rugby teams for seasons 2016–2018). In these countries, unions routinely collect data according to the consensus statement on injury definitions and data collection procedures for studies of injuries in rugby union [14]. Ethical approval for the primary data collections was previously granted in each respective country, noting different data sharing agreements impacted what was able to be provided (see the electronic supplementary material [ESM], Table S1).

### Procedures

Individual player exposure information was collated for every eligible team match in England, Wales, New Zealand, South Africa, and Australia within the study period. The information consisted of a match identification number, player identification number, match minutes, player position, and shirt number. The shirt number identified the player as either a starter (shirt numbers 1–15) or a replacement (shirt numbers 16–23). Individual player data across all team matches were standardised at 80 min (1200 min per team match).

Any replacement player with an exposure of 1 min or longer was included as a replacement in the analysis. A replacement’s entry into the match could have been for any reason including a blood replacement, an HIA replacement, as a front row scrum replacement for a player who had received a yellow card (resulting in 10 min in the “sin bin”), or a permanent injury or tactical replacement. Only the total minutes of exposure were available for each replacement, but this may have been the sum of two distinct appearances within the match, for example, as a blood replacement in the first half and then as a permanent replacement in the second half.

Injury data (from the existing union collections) were compiled by a study coordinator working within each country and provided to the lead investigator of this study. Data included player identification number, playing position, match quarter, body site, match event at the time of injury, and days absence for the injury, noting exclusions of variables in the supplementary file (see the ESM). Any possible identifiers, such as player names, teams, and match dates, were removed prior to sharing, so that the lead investigator could not identify individual players or their injuries. An injury was defined as any injury that resulted in a player being unable to take a full part in future rugby training or match play for more than 24 h from midnight at the end of the day the injury was sustained (time-loss match injury) [14].

### Equality, Diversity, and Inclusion Statement

Our author team comprised four women and 13 men, working in five different countries in both international and national governing bodies and academic institutions. Authors were from clinical and research backgrounds with a range of experience. The study population was exclusively elite male due to the equivalent data being unavailable for sub-elite populations and the women’s game over the same period.

The international governing body (responsible for the laws of the game) and national governing bodies (responsible for administering the game in specific nations) were involved in developing the research questions and providing anonymised data; no players or coaches were directly involved in the research.

### Statistical Analysis

Exposure was calculated from summed minutes of play for individual starting and separately for replacement players across all matches. Injury incidence rates were then determined separately for starters and replacements by dividing the number of injuries sustained by player exposure and multiplying by 1000 to result in an injury incidence rate per 1000 player match hours (with 95% confidence intervals [CIs]) for the respective groups. Injury severity was determined as the mean and median number of days lost per injury with 95% CIs. Mean injury burden was determined from the product of injury incidence and mean injury severity and defined as the number of days lost per 1000 player match hours with 95% CIs (Excel 365).

Comparisons of injury incidence and mean burden rates for replacements versus starters for all injuries, concussion, playing position, match quarter, body region, and injury event were determined using rate ratios and considered significant if their associated 95% CIs (Poisson distribution) did not include 1.0 (Excel 365) [15]. In the event of a significant rate ratio for mean burden, a further comparison was made for median burden, which was calculated using median injury severity and injury incidence. A statistical difference for injury burden between starters and replacements is reported only when the mean and median burden were both significantly different. A generalised linear mixed model with a Poisson distribution, log link, offset for exposure, and random effect for team was used to assess the effect of clustering by team [16, 17]. The impact of clustering on the associated CIs for incidence and rate ratios was shown to be negligible, and so the results of these models are not presented. Starter and replacement injury severity (days lost per injury) was checked for normality of distribution using the Kolmogorov–Smirnov test and subsequently compared using the Hodges–Lehman estimator to derive the median of the differences in severity and the associated 95% CIs (IBM SPSS Statistics, Version 27). Median differences were considered significant if the 95% CIs did not include zero.

Match identification numbers were used to collate injury and exposure data for all matches where both teams were from any one of England, Wales, South Africa, New Zealand, or Australia. To determine associations between the number of replacements used and the total minutes played by replacements, and the count/burden of injuries sustained by the opposing team during a match, generalised linear mixed-effects models were fitted using the *glmmTMB* package [18] in R Statistical Software (v4.1.1; [19]), with a negative binomial distribution and a random effect to account for clustering by team. The resultant beta coefficient (*β*) represented the change in injury count/burden for any given team that was associated with each additional replacement or replacement minute used by the opposing team.

## Results

### Player Exposures

**Fig. 1:**
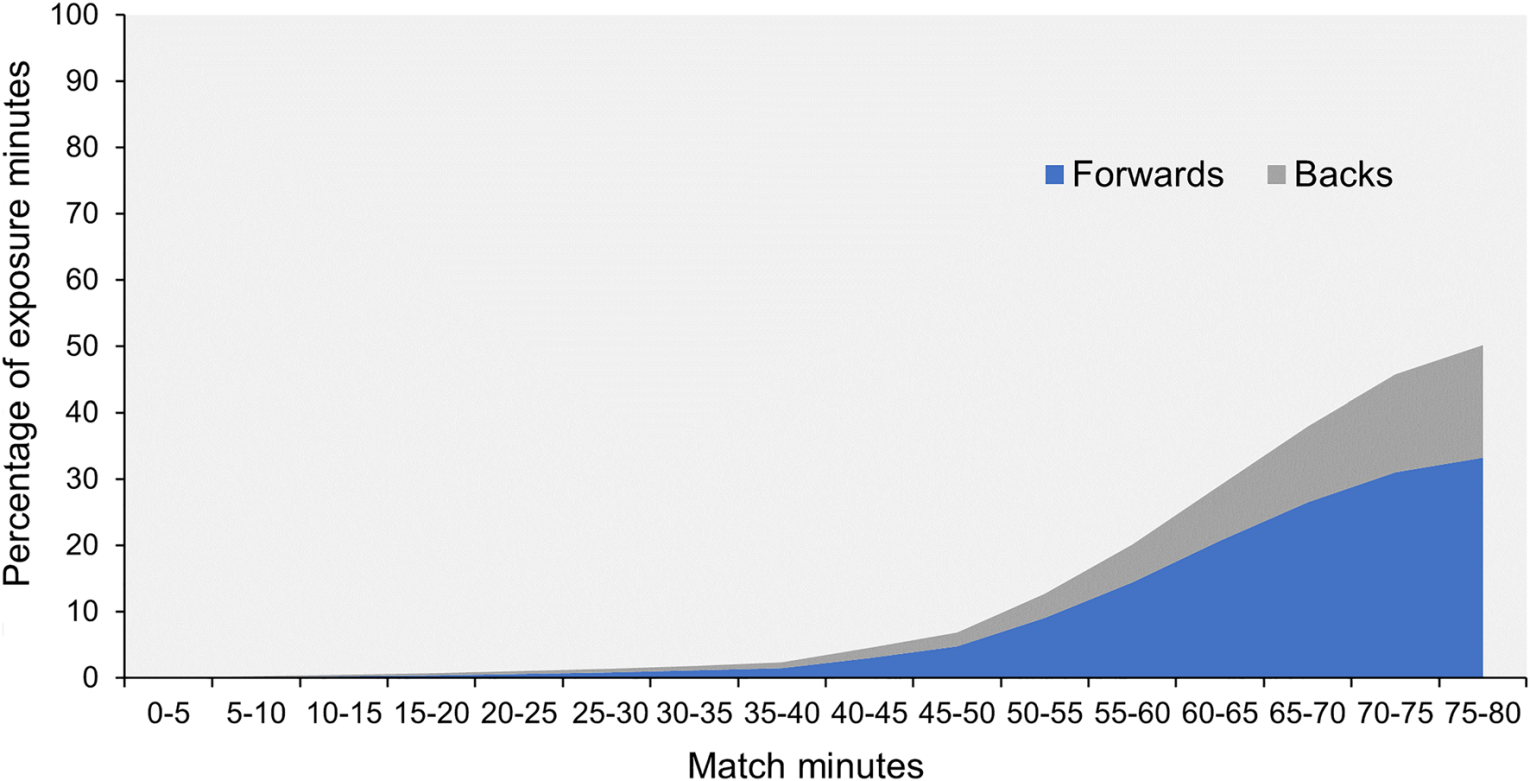
Percentage of exposure minutes for replacement forwards and backs at 5-min match intervals

Out of 35,292 match exposure hours, 4714 h (13.4%) were attributable to replacements, with replacement forwards accounting for 9.2% and replacement backs 4.2% of total exposure. Replacement exposure increased as the match progressed, culminating in 41% of all exposure minutes in the last match quarter (60–80 min) (Fig. 1). In the final match quarter, forward replacements accounted for 52% of the total forwards’ exposure, with back replacements accounting for 27% of backs’ exposure. All eight replacements were used by a team in 54% of matches, seven or more replacements were used in 77% of matches, and six or more replacements were used in 89% of matches. In 71% of matches, a team used between 120 and 200 replacement minutes.

### Replacements in Team Matches and Fixtures

**Fig. 2:**
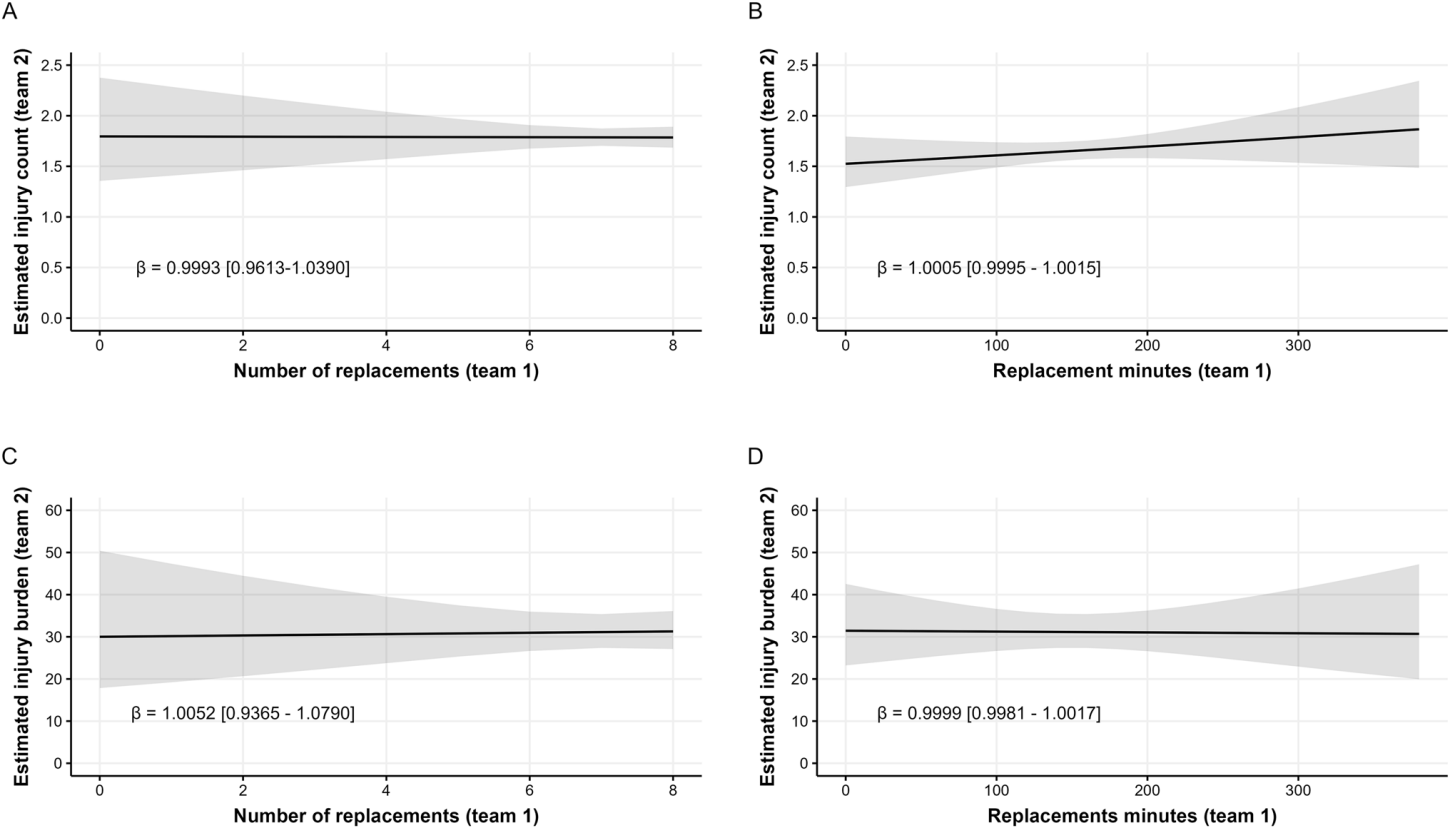
Associations between the number of replacements (**A**, **C**) and replacement minutes (**B**, **D**) used by team 1 and the number of injuries (**A**, **B**) and burden of injuries (**C**, **D**) sustained by team 2 per match. *Shaded areas* represent 95% CIs around the estimate and the resultant beta coefficient (*β*) represents the change in injury count/burden for a team associated with each additional replacement or replacement minute used by the opposing team. *CI* confidence interval

A total of 1224 matches included injury and exposure data for starting and replacement players for both teams. The number of injuries experienced by a team (team 2) was unaffected by the number of replacements used by their opponents (team 1), remaining relatively constant at 1.8 per match across the range from zero to eight replacements (Fig. 2A, *β* = 0.9993, 95% CI 0.9613–1.0390). Similarly, total replacement minutes used by team 1 did not influence the number of injuries sustained by team 2 (*β* = 1.0005, 95% CI 0.9995–1.0015; Fig. 2B).

There was no association between either the number of replacements (*β* = 1.0052, 95% CI 0.9365–1.0790; Fig. 2C) or total replacement minutes (*β* = 0.9999, 95% CI 0.9981–1.0017; Fig. 2D) used by one team in a match and the injury burden sustained by the opposing team in that match.

In 38% of matches, both teams used the same number of replacements, and in 32% of matches, one team used one more replacement than the other.

### Injury Incidence Rates, Severity, and Burden

There was no difference in the overall injury incidence rate between starters (78.2 injuries per 1000 player match hours, 95% CI 75.2–81.4) and replacements (77.0 injuries per 1000 player match hours, 95% CI 69.5–85.3; rate ratio 0.98, 95% CI 0.88–1.10) (Table 1). Median injury severity was higher for replacements (median 14.5 days) compared with starters (median 12 days; median of differences − 2.0, 95% CI − 3.0 to − 1.0). The injury burden calculated for both mean and median burden was greater for replacements (rate ratios: mean 1.31, 95% CI 1.17–1.46; median 1.19, 95% CI 1.06–1.33). There was no overall difference in concussion incidence or median burden between starters and replacements (Table 1).

**Table 1:** Incidence (injuries per 1000 match hours, 95% CI), mean severity (days lost per injury, 95% CI), mean burden (total days lost per 1000 match hours, 95% CI), and median severity (days lost per injury, IQR) for all injuries and concussions in starters and replacements

	All	Starters	Replacements	
**All injuries**	*n* = 2756	*n* = 2393	*n* = 363	Rate ratio (95% CI)
Incidence	78.1 (75.2–81.0)	78.2 (75.2–81.4)	77.0 (69.5–85.3)	0.98 (0.88–1.10)
Mean burden	2632 (2535–2732)	2528 (2429–2631)	3303 (2980–3661)	**1.31 (1.17**–**1.46)**^a^
Mean severity	33.7 (32.5–35.0)	32.3 (31.0–33.6)	42.9 (38.7–47.5)	
				Median of differences (95% CI)
Median severity	12.0 (6.0–36.0)	12.0 (5.0–34.0)	14.5 (6.0–51.3)	**− 2.0 (− 3.0 to − 1.0)**
**Concussion**	*n* = 542	*n* = 479	*n* = 63	Rate ratio (95% CI)
Incidence	15.4 (14.1–16.7)	15.7 (14.3–17.1)	13.4 (10.4–17.1)	0.85 (0.66–1.11)
Mean burden	289 (266–314)	275 (251–301)	381 (297–487)	1.38 (1.06–1.80)
Mean severity	18.8 (17.3–20.5)	17.5 (16.0–19.2)	28.5 (22.2–36.5)	
				Median of differences (95% CI)
Median severity	9.0 (6.0–15.0)	9.0 (6.0–14.0)	11.0 (6.5–18.0)	− 2.0 (− 4.0 to 0.0)

Rate ratios (95% CI) are calculated between replacements compared with starters. Bold rate ratios and median of differences indicate significant differences

*CI* confidence interval, *IQR* interquartile range

^a^Indicates that burden calculated using both mean and median severity is significantly higher in replacements (median burden rate ratio 1.19, 95% CI 1.06–1.33)

### Positional Groups

There was no difference in injury incidence rate between starters and replacements when considered by position (starting forward 89.0, 95% CI 83.6–94.4 vs replacement forward 87.5, 95% CI 73.4–101.6; rate ratio 0.98, 95% CI 0.82–1.19; starting back 81.4, 95% CI 76.9–86.9 vs replacement back 90.6, 95% CI 69.8–111.4; rate ratio 1.11, 95% CI 0.89–1.49). Note, the injury incidence rates reported for forwards and backs are higher than the overall rates because player position data from two countries were unavailable.

Injury severity was higher for forward replacements (median 14.5 days) compared with forward starters (median 11.0 days, median of differences − 2.0, 95% CI − 4.0 to 0.0) and back replacements (median 20.0 days) compared with back starters (median 10.0 days; median of differences − 6.0, 95% CI − 11.0 to − 2.0).

Mean and median burden were higher for forward replacements (mean 3690 days, 95% CI 3093–4287) compared with forward starters (mean 2867 days, 95% CI 2693–3041; rate ratios: mean 1.29, 95% CI 1.08–1.53; median 1.30, 95% CI 1.09–1.54) and for back replacements (mean 3901 days, 95% CI 3006–4796) compared with back starters (mean 2539, 95% CI 2374–2704; rate ratios: mean 1.54, 95% CI 1.21–1.95; median 2.23, 95% CI 1.75–2.82).

### Injury Incidence by Match Quarter

Where timing of injury could be determined (the match quarter was reported as unknown for 16.0 and 12.2% of replacement and starter injuries, respectively), replacements had a lower injury incidence than starters in the third (40–60) and fourth (60–80) match quarters, with replacements being 32 and 22% less likely to be injured than starters in the final two quarters of matches. Median injury severity was higher for replacements than starters in the fourth quarter (Table 2), and mean and median burden were higher for replacements than starters in the second quarter (Table 2; rate ratios: mean 1.74, 95% CI 1.10–2.75; median 2.69, 95% CI 1.70–4.24).

**Table 2:** Incidence (injuries per 1000 match hours, 95% CI), mean severity (days lost per injury, 95% CI), mean burden (total days lost per 1000 match hours, 95% CI), and median severity (days lost per injury, IQR) for all injuries in starters and replacements by match quarter

Match quarter (min)	All	Starters	(*n*)	Replacements	(*n*)	
Incidence (per 1000 match hours in each quarter; 95% CI)						Rate ratio (95% CI)
0–20	47.1 (42.7–51.8)	46.7 (42.4–51.5)	408	100.9 (42.0–242.4)	5	2.16 (0.89–5.21)
20–40	77.5 (71.9–83.6)	77.1 (71.4–83.2)	662	97.4 (62.1–152.8)	19	1.26 (0.80–1.99)
40–60	67.5 (62.3–73.2)	70.1 (64.5–76.2)	550	48.0 (36.3–63.5)	49	**0.68 (0.51–0.92)**
60–80	77.1 (71.5–83.1)	84.2 (76.8–92.3)	456	65.8 (57.8–75.0)	227	**0.78 (0.67–0.92)**
All (known)	67.3 (64.7–70.1)	67.9 (65.0–70.9)	2076	63.6 (56.8–71.3)	300	0.94 (0.84–1.05)

Mean burden (days lost per 1000 match hours; 95% CI)				Rate ratio (95% CI)
0–20	2077 (1886–2287)	2073 (1881–2284)	2925 (1218–7028)	1.41 (0.58–3.41)
20–40	2567 (2382–2768)	2526 (2341–2726)	4401 (2807–6899)	**1.74 (1.10–2.75)**^a^
40–60	2060 (1901–2232)	2119 (1949–2303)	1606 (1214–2125)	0.76 (0.57–1.02)
60–80	2491 (2311–2685)	2242 (2045–2457)	2885 (2533–3285)	1.29 (1.10–1.51)

Mean severity (days lost per injury; 95% CI)			
0–20	44.1 (40.1–48.6)	44.3 (40.2–48.9)	29.0 (12.1–69.7)
20–40	33.1 (30.7–35.7)	32.8 (30.4–35.4)	45.2 (28.8–70.8)
40–60	30.5 (28.2–33.0)	30.2 (27.8–32.9)	33.5 (25.3–44.3)
60–80	32.3 (30.0–34.8)	26.6 (24.3–29.2)	43.8 (38.5–49.9)

Median severity (days lost per injury; IQR)				Median of differences (95% CI)
0–20	17.0 (8.0–52.0)	17.0 (8.0–52.3)	24.0 (3.0–24.0)	5.0 (− 17.0 to 34.0)
20–40	13.0 (6.0–33.0)	12.0 (6.0–33.0)	25.5 (11.3–53.3)	− 7.0 (− 19.0 to 1.0)
40–60	10.0 (5.0–32.5)	10.0 (5.0–31.0)	13.0 (4.8–51.3)	− 1.0 (− 5.0 to 1.0)
60–80	10.0 (5.0–34.0)	9.0 (5.0–27.0)	12.0 (5.0–49.0)	− **3.0 (− 5.0 to** − **1.0)**

Rate ratios (95% CI) are calculated between replacements compared with starters. Bold rate ratios and median of differences indicate significant differences

*CI* confidence interval, *IQR* interquartile range

^a^Indicates that burden calculated using both mean and median severity is significantly higher in replacements (median burden rate ratio 2.69, 95% CI 1.70–4.24)

When injuries in the fourth quarter were subdivided by position, starting forwards had a higher injury incidence rate compared to replacement forwards (103.4, 95% CI 89.3–119.6 vs 68.0, 95% CI 55.1–84.0; rate ratio 0.66, 95% CI 0.48–0.88), but there was no difference between starting and replacement backs (89.1, 95% CI 77.6–102.4 vs 79.8, 95% CI 60.1–105.9; rate ratio 0.90, 95% CI 0.62–1.27).

### Body Region

There were no differences in the injury incidence when considered by specific body regions between starters and replacements (Table 3). There was a significantly higher severity for head/neck injuries for replacements (median 11.0 days) compared with starters (median 9.0 days, median of differences − 2.0, 95% CI − 4.0 to − 1.0). The mean and median burden of lower limb injuries were higher in replacements (mean 1674, 95% CI 1413–1935) compared with starters (mean 1354, 95% CI 1270–1438; rate ratios: mean 1.24, 95% CI 1.05–1.46; median 1.19, 95% CI 1.00–1.40) (Table 3).

**Table 3:** Incidence (injuries per 1000 match hours, 95% CI), mean severity (days lost per injury, 95% CI), mean burden (total days lost per 1000 match hours, 95% CI), and median severity (days lost per injury, IQR) for all injuries in starters and replacements by body region

	All	Starters	(*n*)	Replacements	(*n*)	
	*n* = 2578	*n* = 2240		*n* = 338		
Incidence (per 1000 match hours; 95% CI)						Rate ratio (95% CI)
Head/neck	23.3 (21.6–25.0)	23.5 (21.7–25.3)	630	22.0 (17.4–26.5)	91	0.93 (0.75–1.16)
Upper limb	15.8 (14.4–17.2)	15.8 (14.3–17.3)	423	15.7 (11.9–19.5)	65	0.99 (0.77–1.29)
Trunk	7.0 (6.1–7.9)	7.2 (6.2–8.2)	193	5.8 (3.5–8.1)	24	0.80 (0.53–1.23)
Lower limb	37.2 (35.1–39.4)	37.1 (34.8–39.4)	994	38.1 (32.2–44.1)	158	1.03 (0.84–1.22)

Mean burden (days lost per 1000 match hours; 95% CI)				Rate ratio (95% CI)
Head/neck	488 (452–524)	448 (413–483)	745 (592–898)	1.66 (1.33–2.07)
Upper limb	656 (597–714)	659 (596–722)	634 (480–788)	0.96 (0.74–1.25)
Trunk	109 (95–124)	114 (98–130)	80 (48–112)	0.70 (0.46–1.07)
Lower limb	1397 (1316–1477)	1354 (1270–1438)	1674 (1413–1935)	**1.24 (1.05**–**1.46)**^a^

Mean severity (days lost per injury; 95% CI)			
Head/neck	21.0 (19.4–22.5)	19.1 (17.6–20.6)	33.9 (27.0–40.9)
Upper limb	41.6 (37.9–45.3)	41.8 (37.8–45.7)	40.5 (30.6–50.5)
Trunk	15.6 (13.5–17.6)	15.8 (13.6–18.0)	13.8 (8.3–19.3)
Lower limb	37.5 (35.4–39.7)	36.5 (34.2–38.8)	43.9 (37.1–50.8)

Median severity (days lost per injury; IQR)				Median of differences (95% CI)
Head/neck	9.0 (6.0–17.0)	9.0 (6.0–16.0)	11.0 (6.0–39.0)	− **2.0 (**− **4.0 to** − **1.0)**
Upper limb	17.0 (5.0–56.0)	16.0 (5.0–52.0)	22.0 (5.8–67.0)	− 1.0 (− 5.0 to 3.0)
Trunk	10.0 (4.0–21.0)	10.0 (4.0–21.0)	8.0 (3.8–24.0)	1.0 (− 3.0 to 4.0)
Lower limb	14.0 (5.0–42.0)	13.0 (5.0–41.0)	15.0 (5.0–46.0)	− 1.0 (− 3.0 to 1.0)

Rate ratios (95% CI) are calculated for replacements compared with starters. Bold rate ratios and median of differences indicate significant differences

*CI* confidence interval, *IQR* interquartile range

^a^Indicates that burden calculated using both mean and median severity is significantly higher in replacements (median burden rate ratio 1.19, 95% CI 1.00–1.40^b^)

^b^Lower 95% CI is rounded down from 1.003 to 1.00 and therefore considered significant

### Injury Event

There was no difference in injury incidence between starters and replacements and starters for any match event (Table 4). The tackle (including tackled and tackling combined) had the highest injury incidence, accounting for 49 and 43% of injuries to starters and replacements, respectively. The match event was reported as unknown for 12 and 13% of replacement and starter injuries, respectively. The mean and median burden of injuries when replacements were tackled (mean 858, 95% CI 658–1057) were higher than when starters were tackled (mean 662, 95% CI 608–717; rate ratios: mean 1.30, 95% CI 1.01–1.66, median 1.87, 95% CI 1.46–2.40) (Table 4). Median severity was greater for tackled replacements (median 23 days) compared with tackled starters (median 10 days; median of differences − 4.0, 95% CI − 9.0 to − 1.0).

**Table 4:** Incidence (injuries per 1000 match hours, 95% CI), mean severity (days lost per injury, 95% CI), mean burden (total days lost per 1000 match hours, 95% CI), and median severity (days lost per injury, IQR) for all injuries in starters and replacements by match event

Competition	All	Starters	*n*	Replacements	*n*	
Incidence (per 1000 match hours; 95% CI)						Rate ratio (95% CI)
Tackled	20.5 (18.9–22.1)	21.0 (19.3–22.8)	564	17.1 (13.1–21.1)	71	0.81 (0.64–1.04)
Tackling	20.1 (18.5–21.7)	20.4 (18.7–22.1)	547	18.1 (14.0–22.2)	75	0.89 (0.70–1.13)
Ruck/maul	11.4 (10.2–12.6)	10.9 (9.7–12.2)	293	14.5 (10.8–18.1)	60	1.32 (1.00–1.75)
Scrum	2.2 (1.7–2.7)	2.1 (1.5–2.6)	55	3.1 (1.4–4.8)	13	1.53 (0.84–2.80)
Lineout	0.7 (0.4–1.0)	0.8 (0.4–1.1)	21		1	
Collision	6.6 (5.7–7.5)	6.8 (5.8–7.8)	183	5.3 (3.1–7.5)	22	0.78 (0.50–1.21)
Running/open play	8.9 (7.9–10.0)	8.8 (7.7–10.0)	237	9.4 (6.5–12.4)	39	1.06 (0.76–1.49)
All known	70.5 (67.5–73.4)	70.9 (67.7–74.1)	1900	67.8 (59.9–75.7)	281	0.96 (0.84–1.08)

Mean burden (days lost per 1000 match hours; 95% CI)				Rate ratio (95% CI)
Tackled	689 (635–742)	662 (608–717)	858 (658–1057)	**1.30 (1.01–1.66)**^a^
Tackling	626 (576–675)	615 (563–666)	698 (540–855)	1.13 (0.89–1.44)
Ruck/maul	398 (357–440)	389 (344–434)	459 (343–576)	1.18 (0.89–1.56)
Scrum	63 (48–78)	62 (46–79)	70 (32–108)	1.12 (0.61–2.06)
Lineout	29 (17–42)	31 (18–45)		
Collision	168 (145–192)	155 (132–177)	257 (149–364)	1.66 (1.07–2.58)
Running/open play	315 (278–352)	315 (275–355)	319 (219–419)	1.01 (0.72–1.42)

Mean severity (days lost per injury; 95% CI)			
Tackled	33.6 (31.0–36.2)	31.5 (28.9–34.1)	50.1 (38.4–61.7)
Tackling	31.1 (28.7–33.6)	30.1 (27.6–32.6)	38.6 (29.8–47.3)
Ruck/maul	34.9 (31.3–38.6)	35.6 (31.5–39.7)	31.7 (23.7–39.8)
Scrum	28.8 (22.0–35.7)	30.4 (22.4–38.5)	22.4 (10.2–34.6)
Lineout	41.5 (24.1–58.8)	40.2 (23.0–57.4)	
Collision	25.4 (22.0–28.9)	22.7 (19.4–25.9)	48.4 (28.2–68.6)
Running/open play	35.4 (31.2–39.5)	35.6 (31.1–40.1)	33.9 (23.2–44.5)

Median severity (days lost per injury; IQR)				Median of differences (95% CI)
Tackled	11.0 (5.0–33.0)	10.0 (5.0–32.0)	23.0 (7.5–47.4)	− **4.0 (**− **9.0 to** − **1.0)**
Tackling	11.0 (5.8–31.0)	11.0 (6.0–30.0)	11.0 (5.0–46.5)	− 1.0 (− 3.0 to 2.0)
Ruck/maul	12.0 (5.0–42.0)	11.0 (5.0–37.0)	19.5 (5.8–46.5)	− 1.0 (− 7.0 to 2.0)
Scrum	10.0 (4.0–26.5)	10.0 (3.0–24.0)	14.0 (6.0–36.0)	− 2.0 (− 14.0 to 5.0)
Lineout	26.0 (9.0–43.0)	24.5 (8.3–38.5)		
Collision	9.0 (5.0–27.3)	9.0 (5.0–25.8)	9.5 (5.0–58.8)	− 2.0 (− 10.0 to 2.0)
Running/open play	14.0 (7.0–36.0)	14.0 (7.0–36.5)	12.0 (7.0–27.5)	1.0 (− 3.0 to 6.0)

Rate ratios (95% CI) are calculated for replacements compared with starters. Bold rate ratios and median of differences indicate significant differences. Replacement lineout calculations have been excluded due to a sample of one injury

*CI* confidence interval, *IQR* interquartile range

^a^Indicates that burden calculated using both mean and median severity is significantly higher in replacements (median burden rate ratio 1.87, 95% CI 1.46–2.40)

## Discussion

In this study, the number of match minutes or total number of replacements used by one team was not associated with the number or burden of injuries sustained by the opposing team. The overall match injury incidence rate was similar in starting and replacement players. Starting players had a higher injury incidence rate than replacement players in the third and fourth match quarters, largely attributable to the high rate in starting forwards. The injury severity and burden were greater in replacements compared with starters. In brief, the findings reflect, firstly, the number of replacement players used by one team does not influence the number of injuries sustained by the other team, secondly, a small increase in injury risk for starters compared with replacements towards the end of matches, and, thirdly, that replacements get injured more severely and have a higher injury burden than starters.

### Team Replacements/Injury Analysis

This study did not demonstrate any association between the number of replacements or replacement minutes used by one team and the number or burden of injuries sustained by the opposing team. Therefore, the perception that the more extensive use of replacements by a team results in a greater number of injuries to their opponents was not borne out. It should be considered that this association may be limited by the extensive use of replacements in nearly all team matches, with all eight replacements being used in 54% of matches and at least six replacements being used in 89% of matches. In 70% of matches, there was either no difference in the number of replacements used between the two teams (38% of matches) or only one more replacement was used by one team (32% of matches), and therefore, there were few situations where there would be a large mismatch in the number of replacement players on one team compared with the opposing team. This highlights the fact that the current study describes matches played under replacement laws in professional competitions over seasons 2016/17–2018/19, and we have limited ability to extrapolate the effect of teams being limited to fewer replacements.

### Overall Injury Incidence Rates

Across match play, there was no difference in injury incidence rates between starting and replacement players. A difference in injury incidence rates between starters and replacements was observed in the second half of matches. Several factors could account for this difference, including the effect of fatigue, with overall injury incidence rates also lowest in the first match quarter. However, it should be noted that starters’ injury incidence for all injuries did not continue to increase into the third and fourth quarter (Table 2). Therefore, although there is potential for fatigue increasing injury risk later in the match, it does not appear to follow a linear or ‘dose response’ to accumulated playing time. That the fourth quarter injury incidence for starters (when there is a greater chance of starter–replacement interactions) is only slightly higher than the second quarter suggests that the replacements may only have a limited impact on starter injuries later in the match. The higher injury incidences in the second quarter compared with the first, and fourth quarter compared with the third could be due to player fatigue within each half, but it should also be considered that some injuries may occur earlier than recorded if the injury only manifests later in a match, or if the player attempts to play on whilst injured. This could lead to under-reporting the number of injuries in the first quarter. Evidence regarding fatigue in rugby is limited and relates primarily to player movements/actions; for example, there is evidence of reduced levels of high-intensity activity later in the match [2, 20, 21]. A key aspect of rugby, from both a performance and injury perspective, is the combination of the number and physical demand of contact events. This is difficult to measure but is probably important to consider alongside movement patterns. In relation to this, it has been reported that neither tackler nor ball-carrying proficiency deteriorate with increased player time in match [22]. Therefore, further research into the mechanism of how fatigue affects players and time-to-injury analyses are important.

There are limited data in rugby with which to compare the current findings, but in a study of English Premiership teams in seasons 2002–2004, Brooks et al. [13] reported that starting players had a higher injury incidence in the fourth match quarter compared with replacements [13]. At that time, it was permissible to substitute up to two front row players and up to five other players, which is similar to the possibility of up to eight substitutions during the seasons studied here. However, there is no empirical data to demonstrate how teams utilised replacements in 2002, making comparisons difficult.

It is of note that incidence was higher for the starting forwards in the fourth match quarter compared with replacement forwards, whereas this was not the case for backs. At the professional level, squads of 23 contain 15 starters and eight replacements and, generally, teams select at least five forwards and three backs to comprise their eight replacements, although, on occasion, this is a six and two split. In the current study, forward replacements accounted for 52% of the total forwards’ exposure in the fourth quarter (which is 84% of the available forward replacement minutes, assuming five forwards are normally available), compared with 64% for backs (64% of available replacement minutes for three backs). The perceived higher need to replace forwards, given the greater physical contact to which forwards are exposed [1, 2] and the specific conditioning that forwards receive, points towards the likelihood of greater fatigue in this group, which might contribute to greater fourth quarter injury rates in forwards who started the match.

The overall injury severity was greater for the replacement players, a finding which appears to be largely driven by a significantly higher severity for replacements in the final match quarter. The current study did not ascertain the mechanism or type of injury for starting and replacement players at each match quarter and therefore it is difficult to know the reasons for this result. Lacome et al. (2016) reported that replacement players run at a higher intensity in the first 10 min of coming on compared with the starting player they replaced [23], while Tee et al. (2020) demonstrated that high-speed running was greater for forward replacements compared with starting players [21]. This suggests that in further research, it may be pertinent to include an analysis of starting and replacement player activity intensity at the time of an injurious event to ascertain whether this is associated with the severity of injury. There is also the additional consideration that compared with pre-match warm-up preparation before the start of a match, replacement players may not perform an adequate warm-up prior to entering the match, thus leaving them prone to more severe injuries.

### Strengths and Limitations

A strength of the current study was the collaborative effort to combine data from different countries’ professional teams to answer a pertinent research question. This was achievable due to largely standardised data fields, although it should also be acknowledged that different data collection tools were used across different study sites. There are some limitations to the current study. We did not account for some of the replacement exposures possibly being earlier in the match for blood or HIA replacements, or on some occasions as a scrum front row replacement for a carded front row player. It is unlikely that this would have had a large impact on the distribution of starter and replacement exposures over each match quarter. The match activity or event, as well as the time of injury, are reported by players and medical staff after the injury, and this means that in some cases, neither time nor match activity could be assigned. The match quarter was also not reported for some injuries, and therefore, it is not known whether these unknown injuries are distributed evenly across match quarters.

## Conclusion

The current study is a first step in understanding injuries to starting and replacement players in professional rugby. In this cross-sectional study, the use of replacements by one team did not impact on the injuries sustained by the opposing team. An increased incidence of injury in starting compared to replacement players occurred in the second half, particularly in the forwards. Although fatigue may contribute to these differences, starters were not significantly more likely to get injured in the third or fourth quarter compared with the second quarter. This study provides objective data to the governing body to aid decision-making processes in the area but does not support changes to the current laws on the use of replacements in the game.

### Supplementary Information

Below is the link to the electronic supplementary material.

Supplementary file1 (DOCX 14 kb)

## Declarations

### Availability of data

The data used for this study are not publicly available. Data sharing agreements, between contributing countries and the lead author’s institution, allowed the collation of anonymous data but did not extend to making this publicly available given restrictions from individual country governing bodies.
