# Supplementary material for: Injury in Starting and Replacement Players from Five Professional Men’s Rugby Unions
Source: Sports Med. 2024 Apr 12;54(8):2157–67. doi: 10.1007/s40279-024-02014-3 (PMC11329520; doi:10.1007/s40279-024-02014-3)
Supplement: Supplementary file 1 — Supplementary file1 (DOCX 14 kb) [file 40279_2024_2014_MOESM1_ESM.docx]

**Supplementary file**

**Table S1.** Data provided by each contributing country

|  | England | Wales | New Zealand | South Africa | Australia |
| --- | --- | --- | --- | --- | --- |
| Data field | Premiership | Pro-14 | Super Rugby | Super Rugby | Super Rugby |
| Player Exposure | League match cards | Player-worn GPS | OPTA match cards | OPTA match cards | OPTA match cards |
| Injury severity | Yes | Yes | Yes | Yes | Yes |
| Concussion | Yes | Yes | Yes | Yes | Yes |
| Match quarter | Yes | Yes | Yes | Yes | Yes |
| Playing position | Yes | No | Yes | Yes | No |
| Body region | Yes | Yes | Yes | Yes | No |
| Injury event | Yes | Yes | Yes | Yes | No |
